# Chiluria in a lymphatic filariasis endemic area

**DOI:** 10.1186/s13104-018-3357-y

**Published:** 2018-05-02

**Authors:** Paulo Sérgio Ramos de Araújo, Valter Romão de Souza Junior, Anderson de Souza, Luciana de Barros Correia Fontes, Eduardo Brandao, Abraham Rocha

**Affiliations:** 1 Federal University of Pernambuco, Av. Prof. Moraes Rego 1235, Recife, Pernambuco 50670-901 Brazil; 2 Instituto Aggeu Magalhaes, FIOCRUZ, Av. Prof. Moraes Rego 1235, Recife, 50670-901 Brazil

**Keywords:** Chyluria, Lymphatic filariasis, *Wuchereria bancrofti*

## Abstract

**Objective:**

To establish clinical and laboratory data of individuals presenting chyluria in endemic areas.

**Results:**

75 individuals were studied. The majority were females with an average age of 45 years residing in the Metropolitan Region of Recife. The mean time between the beginning of the presentation of chyluria and the first care service in the Serviço de Referencia Nacional em Filarioses was approximately 5 years. The most frequent urinalysis changes were hematuria (27.6%), leukocytes (21.9%) and proteinuria (10.5%). The Addis test showed mean values of 155.43 E/min/mL of cylinders, 52,892 E/min/mL of erythrocytes and 291,660 E/min/mL of leukocytes. Among recorded cases, proteinuria had a mean value of 1372.80 mg/dL in 24 h, and the presence of lymphocytes in the urine was positive in 68.3%. Among lymphatic filariasis tests, immunochromatography was positive in 16.7%, there was circulating filarial antigen determined by detection of OG4C3 antibodies in 7.7% and microfilaremia in only 1/55.

## Introduction

Lymphatic filariasis (LF), also known as elephantiasis, occurs through infection by *Brugia malayi*, *Brugia timori* and *Wuchereria bancrofti* [1, 2]. It has been considered a neglected disease, responsible for permanent or long-term physical disabilities in more than 40 million people, a considerable portion of a total of 120 million people infected worldwide [3, 4]. Endemic in 72 subtropical and tropical countries, it is estimated that there are 947 million individuals at risk of infection in 54 countries [5–7].

Currently, according to the Pan American Health Organization, there are 720,000 infected people in the American continent, distributed in Guyana, Dominican Republic and Haiti, and 9 million people living in areas with a risk of contamination [8]. In Brazil, there is no record of new autochthonous cases of MCF since 2014, causing the Ministry of Health to start a program of verification and elimination of LF, aiming the interruption of its transmission [9].

Adult worms of *Wuchereria bancrofti* prefer the lymphatic system, where they are able to live from 4 to 8 years [10]. Despite a tropism through lymphatic vessels, the pathogenesis of the damage is still not completely clear. It is known, however, that the presence of adult worms in vessels and lymph nodes, mainly in the pelvic region (legs and scrotum), breasts and arms, causes damage to these structures, leading to lymphedema, hydrocele, keruria and elephantiasis [11–13]. Although it occurs in other clinical conditions, lymphatic fistulation syndromes (lichen, kilocele, and lymphocele) are considered strongly associated with this pathology in endemic areas of LF.

Chyluria, or milky urine, is the presence of pylorus in the urine, a fluid composed of lymph and chylomicrons absorbed by lymphatic vessels, transported to the thoracic duct and then drained into the subclavian vein. Normally, lymphatic vessels do not communicate with the urinary tract. When this occurs, the chylium escapes into the urine, and the point of leakage may occur in the kidney, ureter or bladder. The urine then acquires a characteristically milky coloration [14, 15].

It is believed that approximately 2–10% of individuals with LF develop chyluria, which is the main parasitic etiology of this clinical finding [16–18]. Chyluria generally occurs several years after infection by *Wuchereria bancrofti* and is characteristically intermittent [19]. As it causes protein loss, its association with asthenia, weight loss and malnutrition is common [20].

## Main text

### Methods

#### Study area, population and study design

A retrospective study was carried out, based on data recorded in the records of the National Reference Service in Filariasis of the Aggeu Magalhães Research Center (CPqAM), a unit of the Oswaldo Cruz Foundation (Fiocruz) in the State of Pernambuco, Recife, northeast of Brazil. This service meets the demands of all regions of the country.

There was respect for the universal principles of Human Rights and Bioethics in Research. The project was previously approved by Ethics Committee of Instituto Aggeu Magalhaes. It was considered a census sample of medical records of patients seen between 1996 and 2016.

In the analysis of the data, measurements of central tendency, dispersion measures and statistical tests were obtained, adopting a sampling error of 5%, with the aid of the software Statistical Package for the Social Sciences (SPSS), version 23. The Chi square test, Student’s *t* test, and Fisher’s exact test were used to cross-analyze the variables: gender, 24-h Proteinuria, Leukocytes, Casts, and Blood Cells.

### Results

Among the 75 individuals studied, the majority were females (45/75, 60%), with a mean age of 45.2 years (minimum age of 14 and maximum age of 78 years). The majority (94.7%) lived in the Metropolitan Region of Recife (MRR). In descending order, participants lived in Recife (34 cases, 45.4%), Jaboatão dos Guararapes (13, 17.3%), Olinda (11, 14.7%), Paulista (9, 12%), Camaragibe (2, 2.7%), Cabo de Santo Agostinho (1, 1.3%) and São Lourenço da Mata (1, 1.3%). Among the cities in the countryside of Pernambuco, Vitória de Santo Antão, Caruaru and Riacho das Almas contributed with two (2.7%), one (1.3%) and one (1.3%) cases, respectively. (Table 1).

**Table 1:** Participant characteristics

Characteristics	N	%
Age (years)		
≤ 14	1	1.3
15–30	18	24.0
31–45	20	26.6
46–65	27	36.0
> 65	9	12.0
Gender		
Male	30	40.0
Female	45	60.0
Origen/cities of Pernambuco state		
Recife	34	45.4
Jaboatão dos Guararapes	13	17.3
Olinda	11	14.7
Paulista	9	12.0
Camaragibe	2	2.7
Cabo de Santo Agostinho	1	1.3
São Lourenço da Mata	1	1.3
Vitória de Santo	2	2.7
Caruaru	1	1.3
Riacho das Almas	1	1.3

The time elapsed between the first episode of chyluria and the first care service in the SRNF was on average 74.5 months (minimum time of 1 month and maximum of 456 months). Among the changes in urinalysis most frequently found in medical records, hematuria (27.6%), leukocyte (21.9%), cloudy appearance (14.3%), presence of proteins (10.5%) and deposits (5.7%) were reported. (Table 2).

**Table 2:** Laboratory parameters of urinalysis and LF biomarkers in 75 individuals with Chyluria treated at the SRNF from 1996 to 2016

Parameter	Average value	Variation	p value
Addis test			
Leukocytes	33,058	28.4–140,511	0.3789
Casts	155.43	0–2310	0.479
Blood cells	52,892	0.550–291,660	0.476
Urine lymphocytes (mg/dL)			
Present		32 (63.3%)	
Absent		17 (34.7%)	
Biochemistry			
24-h proteinuria (mg/dL)	1372.80	0.257–14,000	0.387

Tests	Results	Number of reagent samples (%)
Biomarkers		
Microfilaremia	Positive	1 (1.8%)
	Negative	55 (98.2%)
Immunochromatographic test (ICT)	Positive	7 (16.7%)
	Negative	35 (83.3)
OG4C3	Positive	1 (7.7%)
	Negative	12 (92.3%)

The mean value of cells and casts, through Addis test, was 155.43 E/min/mL (minimum and maximum value of 2310 E/min/mL), mean value of red cells was 52,892 E/min/mL (minimum value of 0.550 E/min/mL and maximum value of 291,660 E/min/mL), and mean leukocyte value was 33,058 E/min/mL (minimum value of 28.4 E/min/mL and maximum value of 140,511 E/min/mL). Among the 57 individuals who underwent 24-h proteinuria, the majority (61%) presented changed values, with an average value of 1372.80 mg/dL (minimum value of 0.257 mg/dL and a maximum value of 14,000 mg/dL). Urinary lymphocytes were positive in most cases (68.3%). Regarding the biomarkers for LF, the immunochromatographic test (ICT) was positive in 16.7% of the cases. The presence of circulating antigens OG4C3 was positive in only 7.7% of the cases and microfilaremia was positive in only 1/55 of the individuals.

Among the individuals studied, 45% (34/75) performed ultrasonography (USG) of the abdomen and/or scrotal pouch and/or lower limbs, and half (17/34) showed echographic changes. Diffuse soft tissue lower limb edema, hydrocele, scrotal pouch lymphangiectasis, unilateral renal ectasia, and bladder echoes were the most frequently described changes.

In the reported cases of pain and in relation to its topography 10 patients (13.3% of the sample), there was a significant difference (p < 0.05) between the female sex (30% of those) and male (10%. According to Fisher’s exact test there was also a significant association (p < 0.01) between lymphocyte count and number of erythrocytes in the urine.

### Discussion

In this study, we retrospectively reviewed the medical records of 75 individuals living in an endemic area of LF. Individuals complained of chyluria. There was a slight predominance of women in the sample, while the majority of studies had a higher proportion of men [14, 21–23]. However, this may be related to the fact that women seek more medical care. The mean age of the individuals at the time of the first hospital visit was 45 years of age. In India, Tandon et al. [23] described chyluria in patients with LF in individuals aged 15–30 years [23], and this higher age range in our sample may partly reflect a delay in care.

The diagnosis of LF has been based mainly on the clinical expression of residents of endemic areas. Hydrocele and lymphedema are the main clinical manifestations, whereas chyluria is a less common presentation [24] that manifests itself through milky-color urine associated with flank pain, similar to ureteric cramps [25] and may be triggered by meals with high lipid contents. More severe cases may result in malnutrition, hypoproteinemia, immune dysfunction and hypercoagulability states [26, 27]. Chyluria may present an unpredictable clinical course, including spontaneous remission in up to half of the individuals [28].

Chyluria may still be a manifestation of tuberculosis and deep mycoses, in addition to non-infectious causes such as traumas, post-surgical status such as partial nephrectomy and aorta-iliac by-pass, lymphatic malformations, pelvic tumors, irradiation, pregnancy and malformations such as stenosis of the thoracic duct [29]. All our patients underwent ultrasound examinations of the abdomen, and none of them had obstructive causes or the presence of tumors. However, we were not able to perform other diagnostic tests to rule out other infectious causes.

Some biomarkers have been used for the diagnosis of filarial infections and are able to detect microfilariae and/or their antigens in peripheral blood, besides the possibility of using ultrasound to identify adult worms in lymphatic vessels [30]. Among the most common diagnostic tests, the identification of microfilariae in the peripheral blood, identification of *W. bancrofti* antigens by an immunoenzymatic assay based on Og4C3 monoclonal antibodies and immunochromatographic test (ICT) are highlighted [31–33]. The ICT card test is a fast test performed by digital capillary punctures where total blood is spread on a card impregnated with filarial antigen markers, and the result is interpreted by colorimetric reaction. Despite the easy execution of the test, a negative result is not able to exclude filarial infection as a cause of a chronic pathology, since filarial antigens eventually become undetectable in a treated infection even in a scenario of already installed lymphatic damage [34]. In the studied population, only one individual had microfilariae in the peripheral blood. Among those who underwent ICT and Og4C3, only 16.7 and 7.7% presented positive results, which corroborates with other studies describing a low sensitivity of such tests in a scenario of late filarial infection and already installed filarial morbidity [35].

The analysis of urine in patients with chyluria may reveal the presence of chylomicrons and triglycerides. The quantification of these elements is the most specific and sensitive marker for diagnostic confirmation [36]. In our cases, the presence of hematuria (27.6%), possibly due to the deposition of immunocomplexes in the basal glomerular membrane and leukocytes (21.9%), was observed through the Addis test. There was also expression of an abnormal connection between the lymphatic system and the urinary system [37]. Urine lymphocyte screening was present in 63.3%, which is compatible with data from the literature. Some studies have indicated the presence of lymphocytes in the urine as an important microscopic marker due to the formation of venolymphatic fistulas leading to an increased pressure in lymphatic vessels [38–40]. Protein concentration in the 24-h urine test presented mean values of 1372.80 mg/dL, which may reflect a picture of nephritis by immune complex deposition. However, this was not confirmed [41].

## Limitations

- The low proportion of individuals with chyluria and residents of endemic LF areas with registered protocols is a possible limitation of this study.
- We were not able to perform other diagnostic tests to rule out other infectious causes.

## Abbreviations

ICT: immunochromatographic test
FIOCRUZ: Osvaldo Cruz Foundation
